# Ecological effects of ocean acidification and habitat complexity on reef-associated macroinvertebrate communities

**DOI:** 10.1098/rspb.2013.2479

**Published:** 2014-01-22

**Authors:** K. E. Fabricius, G. De'ath, S. Noonan, S. Uthicke

**Affiliations:** Australian Institute of Marine Science, PMB 3, Townsville, Queensland 4810, Australia

**Keywords:** carbon dioxide, pH, habitat quality, reef structural complexity, scleractinian coral cover, reef-associated macroinvertebrate fauna

## Abstract

The ecological effects of ocean acidification (OA) from rising atmospheric carbon dioxide (CO_2_) on benthic marine communities are largely unknown. We investigated *in situ* the consequences of long-term exposure to high CO_2_ on coral-reef-associated macroinvertebrate communities around three shallow volcanic CO_2_ seeps in Papua New Guinea. The densities of many groups and the number of taxa (classes and phyla) of macroinvertebrates were significantly reduced at elevated CO_2_ (425–1100 µatm) compared with control sites. However, sensitivities of some groups, including decapod crustaceans, ascidians and several echinoderms, contrasted with predictions of their physiological CO_2_ tolerances derived from laboratory experiments. High CO_2_ reduced the availability of structurally complex corals that are essential refugia for many reef-associated macroinvertebrates. This loss of habitat complexity was also associated with losses in many macroinvertebrate groups, especially predation-prone mobile taxa, including crustaceans and crinoids. The transition from living to dead coral as substratum and habitat further altered macroinvertebrate communities, with far more taxa losing than gaining in numbers. Our study shows that indirect ecological effects of OA (reduced habitat complexity) will complement its direct physiological effects and together with the loss of coral cover through climate change will severely affect macroinvertebrate communities in coral reefs.

## 1. Introduction

For marine ecosystems, the consequences of long-term exposure to rising carbon dioxide (CO_2_) concentrations are poorly understood. The partial pressure of CO_2_ (*p*CO_2_) in seawater is now higher than it has been for at least 800 000 years and has already reduced global mean seawater pH by 0.1 units [1], a process called ocean acidification (OA). If anthropogenic CO_2_ emissions continue unabated, *p*CO_2_ will increase to two to three times pre-industrial values towards the end of this century [2], reducing global mean pH by up to 0.3 units (a 150% increase in hydrogen ions) and halving carbonate ion concentrations [1–3].

Controlled experiments are essential to identify physiological responses, tolerance curves and thresholds to OA. Such laboratory experiments are being carried out on a rapidly increasing number of species in one or several of their life stages [4–6]. However, experiments have a limited capacity to predict *in situ* ecosystem responses to increasing CO_2_. Some taxa that perform poorly at high CO_2_ under laboratory conditions may be able to acclimatize to high CO_2_ in the longer term. Others that perform well under experimental CO_2_ exposure may be at risk during life stages that have not been experimentally assessed [5,6] or may be indirectly affected by OA-induced ecological changes in habitat properties (e.g. microbial surface biofilms and coralline algae), food webs, competition, diseases and/or community structures [7–9].

Shallow submarine CO_2_ seeps are among the few sites in the world within which the ecological consequences of long-term (multi-decadal) exposure to high CO_2_ can be assessed *in situ*. A small number of such CO_2_ seep systems have been investigated to date, including temperate rocky-shore communities in the Mediterranean Sea [8,10,11], a tropical coral reef in Japan [12], a Mexican groundwater spring system where brackish water high in CO_2_ and alkalinity emerges into coral reefs [13], and three volcanic CO_2_ seeps within coral reefs in Papua New Guinea (PNG) [7].

**Figure 1.**
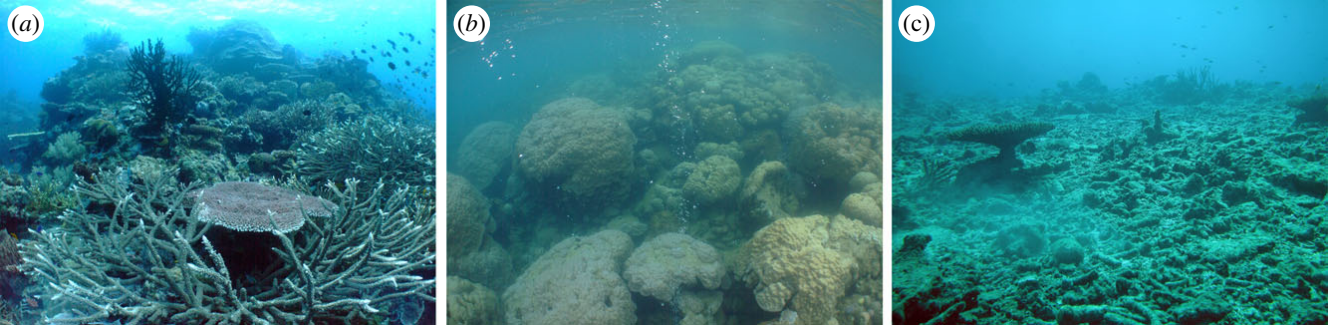
Indo-Pacific reef communities with contrasting structural complexity. (*a*) Structurally complex reef (complexity rating value 4–5) with branching, tabulate and foliose coral morphologies. (*b*) Structurally simplified communities dominated by massive coral morphologies (complexity 2–3), as found in areas of chronic disturbance as shown here from one of the PNG CO_2_ seeps. (*c*) Flattened reef lacking three-dimensional structure (complexity 0–1), as found after major disturbance. (Online version in colour.)

The ecological consequences of OA are likely to be most pronounced in ecosystems built by calcifying biota. The existence of coral reefs depends on the skeletal growth of scleractinian corals, coralline algae and other calcifying organisms, which ‘hypercalcify’ at low CO_2_ owing to the three to fourfold carbonate supersaturation state in tropical seawaters, accreting several kg m^−2^ yr^−1^ of calcium carbonate. In healthy coral reefs, structurally complex branching, tabulate and foliose morphologies are abundant (figure 1*a*), and provide important habitat and refugia for reef-associated organisms [14,15]. However, OA and climate-change-related disturbances (e.g. coral bleaching or severe storms) will increasingly affect the survival and growth of scleractinian corals, with the more sensitive structural corals likely to be most severely affected [16,17]. Coral reefs may therefore shift towards two contrasting structural forms. First, chronic exposure to elevated CO_2_, upwelling or pollution typically leads to structurally simplified coral communities dominated by massive growth forms (often the tough and persistent boulder corals of the genus *Porites* spp.; figure 1*b*), whereas the more sensitive structurally complex branching corals are under-represented [7,13]. Such communities may have high coral cover but low structural and taxonomic diversity. Second, episodic and severe disturbances can lead to widespread coral mortality and ‘reef flattening’ (i.e. the loss of three-dimensional habitat structure; figure 1*c*), which may persist for years to decades after such disturbances [16]. Again, structurally complex corals are more sensitive to these disturbances than massive growth forms [18], further simplifying habitat structures.

Coral reefs are estimated to house 600 000 to more than 9 million species [19], a large proportion of which are macroinvertebrates. For reef-associated fishes, the availability of refugia, both ‘permanent’ (e.g. holes in reef substrata) and ‘transient’ (thickets of living coral branches), is known to increase local species richness [20], and the loss of habitat complexity and coral cover detrimentally affects their communities [15]. For the far more speciose macroinvertebrates, the consequences of losing habitat complexity and live coral remain largely unknown, and the few studies of this topic have resulted in contrasting conclusions. Some documented reduced diversity but unaltered [21] or reduced densities [22] with the erosion of reef framework. Others reported higher cryptofauna diversity and densities on reefs that had lost their three-dimensional habitat structure and living coral cover [23]. Given that the majority of these macroinvertebrates are relatively small-bodied and vulnerable to predation, ecological theory predicts that loss of habitat structure and refugia would have ramifications for the abundance and diversity of their communities [24].

In this study, we investigate the consequences of long-term *in situ* exposure to elevated seawater CO_2_ on benthic macroinvertebrate communities. We compare densities and taxonomic richness of mobile and sessile macroinvertebrate communities along gradients of habitat complexity at the CO_2_ seeps of PNG and adjacent control sites, on patches occupied by live coral and on substrata devoid of living corals. Our results demonstrate some of the mechanisms of how both direct physiological responses and indirect effects of OA may co-determine future macroinvertebrate communities in a high-CO_2_ world.

## 2. Material and methods

### (a) Study sites and seawater chemistry

Studies were conducted at three shallow submarine volcanic CO_2_ seeps and three adjacent control sites with similar geomorphology, seawater temperature and salinity, in Milne Bay Province, PNG [7]. The three seep locations (Dobu, Esa'Ala and Upa Upasina) are located along an active tectonic fault line, and almost pure CO_2_ gas has been streaming through the reef substrata for an unknown period of time (confirmed for approx. 70 years, but possibly much longer [7]), resulting in locally reduced seawater pH. Areas of intense seeping with a median pH_total_ < 7.7 (approx. 1100 µatm *p*CO_2_) were not included in the surveys, because no reef development is found beyond this apparent threshold. Mean hard coral cover was similar at the seeps compared with the adjacent control sites (33% ± 2.4 s.e. versus 31% ± 3.4 s.e.). However, at high CO_2_, the cover of massive *Porites* corals is twice that of the control sites, the cover of structurally complex corals with branching, foliose and tabulate growth forms is reduced threefold, and coral diversity is reduced by 39% [7]. Seawater temperature and salinity are similar between seep and control sites [7].

A total of 968 discrete seawater samples of pH were taken at the six sites from approximately 0.5 m above the benthos (see electronic supplementary material). The data include all samples collected between 2010 and 2012, representing a range of tidal, irradiance and wave conditions to characterize the ranges encountered by the organisms. The samples were immediately analysed for pH, temperature and salinity, and a subset of 450 samples were preserved for later determination of total alkalinity and dissolved inorganic carbon. Other relevant seawater carbonate parameters (aragonite saturation state, dissolved inorganic carbon, *p*CO_2_) were calculated from pH, total alkalinity, dissolved inorganic carbon, salinity and temperature using the R program Seacarb v. 2.4.8 [25].

### (b) Survey methods

Surveys were based on intensive searches along belt transects at the high-CO_2_ and control sites, excluding specimens less than 1 cm in size and fauna within deep crevices that would have required destructive sampling (cryptofauna and infauna). Invertebrates were classified into 46 operational taxonomic units (OTUs), including all major calcifying and non-calcifying groups of mobile macroinvertebrates more than 1 cm in size, and many sessile groups, and comprised a total of 4910 organisms (see electronic supplementary material, table S1). Additionally, 29 OTUs within the echinoderm classes Echinoidea, Holothuroidea and Asteroidea were recorded to the highest possible taxonomic level. PNG is located within the most speciose marine biogeographic region on the Earth (the ‘Coral Triangle’), yielding a large proportion of extant marine species (e.g. estimates for decapod crustaceans: approx. 15 000 species; gastropods: approx. 30 000 species). As the majority of taxa are rare, undescribed or in need of taxonomic revision, OTUs were essential for non-destructive sampling, and groupings were defined to permit consistent *in situ* identification. Taxa were later grouped into phyla and classes (7 and 12 of which were represented in the surveys, respectively), into mobile versus sessile groups, and calcifying versus non-calcifying groups (see electronic supplementary material, table S1).

Thirty-five fine-scale surveys were conducted during two ship-based expeditions to these remote sites (20 surveys in April 2011 and 15 surveys in April 2012). The surveys were conducted at night (more than 1.5 h after the onset of darkness) because many mobile invertebrates remain cryptic during the day. Transect tapes were laid parallel to the slope at 3 m depth. The surveys were conducted within a belt of 20 × 1 m, with a 10 m gap between replicate surveys. Additionally, the larger echinoderm OTUs were recorded along belts 2 m wide along the 50 m length of the two replicate 20 m transects plus gap (total area of 100 m^2^) during both expeditions. During the fine-scale surveys in 2012, the 20 m transects were subdivided into 0.5 × 0.5 m^2^ quadrats (80 quadrats of 0.25 m^2^ per transect), and data were recorded separately for each quadrat. These data also included visual estimates of fine-scale complexity of the reef substrata (six levels [26]; figure 1; electronic supplementary material, tables S1 and S2) and dominant (more than 50% coverage) substratum types: ‘HC’ (living hard corals and *Millepora*), ‘DS’ (substrata devoid of macrofauna and macroflora, but dominated by coralline/turf algae growing on dead coral or coral rubble) or ‘other substrata’. Only 69 of the 1200 quadrats were dominated by ‘other’ substrata, and these were excluded from the second set of analyses.

### (c) Statistical analyses

The analyses were based on two sets of data: (*a*) the combined 2011 and 2012 datasets (*n* = 35 surveys) were used to assess CO_2_ effects only; (*b*) the 2012 data (*n* = 1131 quadrats from 15 surveys) were used to assess the joint effects of CO_2_, complexity and substratum.

For (*a*), generalized linear mixed models (GLMMs) were used to assess the effects of CO_2_ [27]. The models included fixed effects of CO_2_, and random effects of location (Dobu, Esa'Ala and Upa Upasina) and transects within location. The significances of CO_2_ effects were estimated using quasi-*F* tests [28]. Residual plots were used to assess the mean–variance relationships. For the number of OTUs, classes and phyla per quadrat, the models comprised a log link function and variance proportional to the mean (quasi-Poisson) in order to account for over-dispersion. For densities, the variance increased more rapidly with mean values, and there were also large proportions of absences, thus each response was modelled using a negative binomial GLMM [29].

For (*b*), the analyses included the joint effects of CO_2_, complexity and substratum type on the biotic responses. The densities of the main groupings and the number of taxonomic classes (all per 0.25 m^2^) were analysed using GLMMs as for (*a*), but the predictors were CO_2_ and substratum type (categories), and complexity (numeric). The latter was based on quadratic trends across its six levels owing to the nonlinearity of responses. There was no support for higher-order trends across the six levels of complexity. Although CO_2_ and substratum were well balanced, complexity was not well balanced with either CO_2_ or substratum (see electronic supplementary material, table S2), and hence the interactions between the profiles of complexity and each of CO_2_ and substratum were not precisely estimable. First-order interaction effects between CO_2_, substratum and complexity were small and non-significant when adjusted for multiple comparisons across the responses. Hence, we only included main effects of CO_2_, substratum and complexity. These results are presented as partial effects plots and analyses of deviance. All statistical analyses used the statistical software R [30].

## 3. Results

### (a) Seawater chemistry

At the three high-CO_2_ sites, median seawater pH values were 7.95, 7.81 and 7.72 (total scale), *p*CO_2_ concentrations ranged from 441 to 998 µatm, and aragonite saturation state (*Ω*_arag_) ranged from 3.7 to 2.1 (table 1; electronic supplementary material, figures S1 and S2). By contrast, at the three adjacent control sites, the median pH ranged from 8.02 to 7.98 units, *p*CO_2_ from 346 to 413 µatm, and *Ω*_arag_ from 4.2 to 3.7.

**Table 1.** Seawater carbonate chemistry at the three CO_2_ seep and control locations. Medians, and 5th and 95th percentiles, of measured pH, total alkalinity (TA) and salinity, and calculated concentrations of dissolved inorganic carbon (DIC), partial pressure of carbon dioxide (*p*CO_2_) and aragonite saturation state (*Ω*_arag_) are shown (see also [7]; electronic supplementary material, figures S1 and S2).

location	site	pH	TA (µmoles kg^−1^)	DIC (µmoles kg^−1^)	*p*CO_2_ (µatm)	*Ω*_arag_	salinity
Dobu	control	8.01 (7.91,8.10)	2235 (2221,2293)	1942 (1924,1924)	368 (279,558)	4.16 (3.07,4.35)	34.8 (33.8,36.0)
Dobu	high CO_2_	7.72 (7.08,7.99)	2295 (2260,2326)	2147 (1962,1962)	998 (423,3541)	2.10 (0.65,3.75)	34.8 (34.0,36.0)
Esa'Ala	control	8.02 (7.94,8.04)	2262 (2247,2290)	1967 (1885,1885)	413 (258,499)	3.74 (3.34,4.86)	34.8 (34.5,35.0)
Esa'Ala	high CO_2_	7.95 (7.83,8.04)	2291 (2224,2304)	1995 (1947,1947)	441 (257,762)	3.66 (3.15,5.07)	34.8 (34.5,35.0)
Upa-U	control	7.98 (7.91,8.09)	2261 (2225,2326)	1982 (1947,1947)	346 (302,653)	4.08 (2.82,4.57)	34.8 (34.5,36.0)
Upa-U	high CO_2_	7.81 (7.28,8.01)	2308 (2240,2378)	2049 (1964,1964)	624 (410,1564)	2.89 (1.39,3.59)	34.8 (33.8,35.9)

### (b) CO_2_ effects

**Figure 2.**
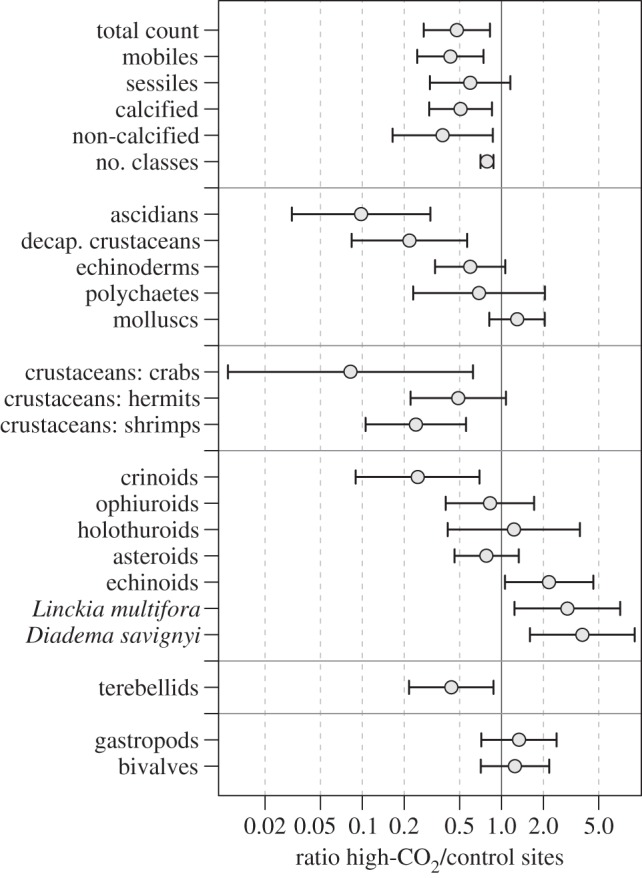
Ratios of the densities or number of taxonomic units of macroinvertebrates at high-CO_2_ relative to control sites (*n* = 35 surveys). Circles mark the estimated ratios, error bars show lower and upper 95% CIs (see electronic supplementary material, tables S5 and S6). Differences are significant (*p* < 0.05) if the error bars do not include the value 1.0.

The total density of macroinvertebrates and the number of taxa per transect were significantly reduced at the high-CO_2_ compared with the control sites (figure 2; electronic supplementary material, table S3). Total density was reduced to 48% of control values (from 10.9 to 5.2 individuals m^−2^), whereas the number of OTUs, classes and phyla were reduced to 77%, 79% and 85%, respectively. Losses at high CO_2_ were significant in both the groupings of calcifying and non-calcifying invertebrates (down to 51% and 38%, respectively). When taxa were grouped by their mobility, a general trend emerged despite the variability between taxa. The total density of the combined mobile groups was strongly reduced at high CO_2_ (down to 43% of control values), whereas that of the combined sessile groups was not significantly different. Of the mobile taxa, the most severely affected groups were the decapod crustaceans (down to 22%) and feather stars (Crinoidea, down to 25%; figure 2; electronic supplementary material, table S3). By contrast, the sea urchins (Echinoidea) increased in density, especially *Diadema savignyi* and *Echinothrix* spp., which are often found around massive *Porites* and other boulder-shaped substrata with low structural complexity. Total sea star (Asteroidea) density was similar at the seeps and controls; however, when *Linckia multifora* was excluded (a species with three times higher densities at the seeps compared with the controls), the density of the remaining asteroids was approximately 50% of control values. Of the sessile invertebrates, the most severely reduced taxa were the sea squirts (Ascidiacea, 22% of controls) and terebellid polychaetes (44%). Mean densities of date mussels (*Lithophaga* spp.) and vermetid gastropods, which live embedded within living massive *Porites* and other substrata, increased approximately 50-fold and eightfold at high CO_2_, respectively, but differences were not statistically significant owing to high variability. In other taxonomic groups, densities were similar at the high-CO_2_ and control sites (see electronic supplementary material, table S3).

### (c) Combined effects of CO_2_, structural complexity and substratum type

The 2012 survey data confirmed that fine-scale structural complexity of the reef habitat was significantly reduced in the transects at the high-CO_2_ compared with control sites (*F*_1,9_ = 24.2, *p* < 0.001; electronic supplementary material, figure S3 and table S4), with similar losses of complexity at all three locations (*F*_2,9_ = 3.4, *p* = 0.078), and non-significant interactions between locations and CO_2_ (*F*_2,9_ = 0.61, *p* = 0.570). The proportions of quadrats with live HC and those with substrata devoid of macrofauna (DS) were similar at high-CO_2_ compared with control sites (53% HC at high CO_2_ versus 49% HC at controls; *n* = 1131, *χ*^2^ = 1.93, *p* = 0.16; electronic supplementary material, table S2).

**Figure 3.**
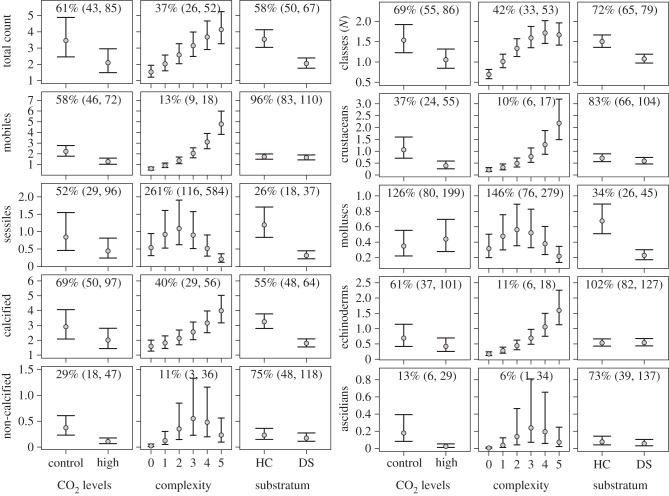
Partial effects plots showing the relationships of macroinvertebrates to CO_2_, complexity and substratum type (HC: quadrats dominated by living hard corals; DS: substrata devoid of macrofauna). Circles indicate estimated marginal mean densities or number of taxonomic classes per 0.25 m^−2^ (total counts = all organisms combined; mobile/sessile and calcifying/non-calcifying = densities of all mobile/sessile and calcifying/non-calcifying organisms combined). Error bars are lower and upper 95% CIs. Estimated percentage change (high CO_2_ to control, complexity rating = 0 to complexity rating = 5, and DS to HC) and their 95% CIs are shown in the panels.

Differences in CO_2_, complexity and substratum type each accounted for large proportions of variation in the macroinvertebrate communities (figure 3*a*,*b*; electronic supplementary material, tables S4 and S5). Partial effects analyses showed that the total density of all macroinvertebrates combined not only declined at high CO_2_ to 61% of control values, but additionally declined to 37% at low compared with high complexity, and to 58% on DS compared with HC. Similarly, the number of classes per quadrat declined to 69% at high CO_2_, to 42% from high to low substratum complexity, and to 72% in quadrats dominated by DS compared with those dominated by HC. The densities of all mobile macroinvertebrates combined (of crinoids, decapod crustaceans, ascidians and terebellid polychaetes) were well explained by both direct CO_2_ effects and the loss in complexity, while substratum type was less important for these groups. Overall, there were significant losses at high CO_2_ in 12 of 22 responses (densities and number of taxa for which effects were estimable; electronic supplementary material, table S5), and increases in one response (Asteroidea). Additionally, 14 of the 22 responses significantly declined with declining complexity, suggesting strong indirect effects of OA on macroinvertebrate communities (figure 3*a*,*b*). For example, crustacean and echinoderm densities were reduced to 10 and 11% in low-compared with high-complexity quadrats. Four other responses (total density of sessile organisms, bivalves, polychaetes and sabellids) had higher densities at low levels of complexity, and several responses had highest values at intermediate complexity. Finally, 14 of the responses also showed significant differences between substratum types, with all but two (Asteroidea and Echinoidea) having reduced densities (18–68%) and number of taxa (72–74%) on DS compared with HC.

## 4. Discussion

### (a) Physiological and ecological effects of ocean acidification

This study demonstrates that OA exerts both direct and indirect effects on tropical marine macroinvertebrate communities in their natural environment. To date, few tropical macroinvertebrates in few life stages have been investigated for their physiological sensitivity to OA, and sensitivities can vary substantially between species. Nevertheless, reviews and meta-analyses of the existing physiological studies of temperate and tropical taxa have supported general predictions about the OA sensitivities of phyla, classes or orders [5,6,31–33]. A comparison of OA sensitivities derived from these reviews with the *in situ* responses observed at the PNG and Mediterranean seeps shows some commonalities, yet also several notable contrasts (table 2), as summarized below.

**Table 2.** Predicted and observed responses of macroinvertebrates to OA. Predictions are based on the generalized main conclusions of meta-analyses and reviews of physiological laboratory experiments [5,6,33]. Observed *in situ* responses are from the CO_2_ seeps in PNG (this study), and the Mediterranean at a pH of more than 7.7 [8,10,11]. Thick arrows represent widespread responses from many taxa; thin arrows represent data from fewer taxa; direction of arrows represents increase (⇑, ↑), little difference (⇔, ↔) or decline/greater reduction (⇓, ↓).

	predicted response (laboratory)	observed *in situ* response (CO_2_ seeps)	
		PNG	Mediterranean
decapod crustaceans	⇔ ↓	**⇓**	**⇓**
molluscs	↔ **⇓**	⇔	⇔
echinoderms	↔ **⇓**	↑ ⇔ ↓	↑ **⇓**
polychaetes	↔ ↓	↔ ↓	↔ **⇓**
ascidians	↑	**⇓**	
mobile groups (cf*.* sessile groups)	**⇑**	**⇓**	
calcifying groups (cf. non-calcifying groups)	**⇓**	⇔	

For decapod crustaceans, our field data have documented severe losses at high CO_2_. By contrast, most physiological laboratory studies predict this aerobic mobile group to be highly CO_2_-tolerant, with negative effects typically only at very high CO_2_ concentrations [4–6], although new studies suggest greater sensitivities in some species [34]. For molluscs, we found no significant *in situ* changes in total densities and the densities of most specific OTUs, whereas experiments suggest either weak or detrimental OA effects on benthic molluscs [4–6,31,33,35]. For echinoderms, our field data also showed only minor changes at high CO_2_ in most classes, while laboratory experiments have typically predicted echinoderms (and, as for molluscs, especially their larvae) to be relatively OA-sensitive, attributable to their highly soluble high-magnesium calcite skeletal structures [5,6,31,33,36] (but see also [32]). Crinoids were the only echinoderm class showing a steep decline. We are not aware of any laboratory study on this class but note that they were decimated during the high-CO_2_ period marking the Permian to Triassic transition. The observation that molluscs and echinoderms appear to be often most OA-sensitive as larvae [5,33] may partly explain the weak responses at the seeps, where most organisms get exposed to high CO_2_ only after settlement. All mollusc and echinoderm OTUs that increased at the seeps (*Lithophaga* spp., Vermetidae, *D. savignyi*, *Echinothrix* spp. and *L. multifora*) are often associated with massive *Porites*, suggesting their densities to be more constrained by habitat availability than by post-settlement exposure to pH at levels above 7.7. Polychaetes are a diverse class comprising calcifying and non-calcifying groups, sessile and mobile groups, and many trophic levels. Not surprisingly, results are mixed for both field and experimental data on their OA tolerance. At the seeps, the Terebellidae showed steep declines at high CO_2_, while the total density of polychaetes (and specifically those of the Sabellidae, despite their common association with *Porites*) remained similar. In the laboratory, responses range from reduced calcification and metabolic depression to high CO_2_ tolerance [37,38], but too few taxa have been investigated for such a diverse group to draw any general conclusions. Finally, ascidians are soft-bodied sessile filter-feeding tunicates, with their tunics made of cellulose-like polysaccharides and proteins. While the field data showed severely reduced ascidian densities at high CO_2_, the few existing laboratory experiments all showed positive survival and developmental responses [33].

There were also substantial differences between field and laboratory results based on the groupings by mobility and calcification (table 2). At the seeps, the decline of the total density of all mobile organisms combined was far greater than that of sessile organisms, whereas calcifying organisms had similar losses in density compared with non-calcifying organisms. By contrast, physiological data predict the most active aerobic mobile groups to be the physiologically most OA tolerant groups owing to effective acid–base regulation of their body fluids, and conclude calcification to be one of the most sensitive responses to OA [5,6]. These discrepancies are not surprising because the groupings by mobility and calcification are composites of diverse taxonomic groups. Nonetheless, the data show that on their own, the physiological mechanisms developed to support mobility and calcification do not render organisms in the field more or less OA-resilient than their sessile and non-calcifying counterparts.

While there are some distinct contrasts between the outcomes of physiological and field studies, a comparison with macroinvertebrate data from the Mediterranean CO_2_ seeps shows many commonalities (table 2). Mediterranean sites with less than 7.7 pH were not considered for this comparison, and the rocky-shore communities of the temperate Mediterranean seeps do not face the issue of loss in structural complexity nor the loss of living coral as substrata. At the Mediterranean seeps, decapod densities were reduced to approximately 40% of control values [8], and there were no major changes in gastropod and bivalve densities [8,11]. Twenty of the 28 more common Mediterranean polychaete OTUs declined in abundances towards higher CO_2_ [8], with the most insensitive species showing genetic adaptation and/or physiological acclimatization [39]. The main contrast between the two regions was a high variability in echinoderm responses in PNG, while most Mediterranean echinoderms appear to be severely affected [11], although a few more CO_2_-tolerant echinoderms are now also being reported [40].

Despite our strong results, certain limitations in the use of CO_2_ seeps as OA proxies should be noted. First, the *p*CO_2_ range at the seeps is more variable than in open ocean systems, with unknown physiological consequences. However, *p*CO_2_ fluctuations only slightly lower than those at the seeps are also being reported from some coastal systems, lagoons or embayments, in zones of upwelling and within metabolically active photosynthetic communities [41]. Second, seeps comprise open populations (i.e. although all sessile and many mobile individuals have had a post-settlement lifetime of exposure to high CO_2_, exposure commences only during larval settlement, and only few taxa with poorly dispersive larvae may have multi-generational exposure). Many of the abundant small crabs, shrimps and crinoids are relatively stationary, with high affinities to specific coral heads or patches of substrata, but larger mobile taxa may have immigrated to the seeps later in their life. Third, seawater temperatures at the seeps represent present-day rather than future conditions, with thermal stress expected to worsen the OA effects [6]. Fourth, the reported associations are not all the effects of CO_2_ and habitat complexity, as additional factors will contribute to the ecological outcomes, such as CO_2_-induced reduced settlement from the loss of crustose coralline algae or altered microbial biofilms. These limitations have to be balanced against the limitations of laboratory perturbation experiments, most of which do not provide for long-term acclimatization nor capture the ecological changes that await organisms in their natural environment as CO_2_ levels continue to rise. Furthermore, responses at higher taxonomic levels (classes or phyla) do not necessarily indicate responses of specific taxa within those classes. For example, the echinoderm data that contained species-level information demonstrated that the apparent lack of response in the class Asteroidea was probably masked by the threefold increased densities of a single species (*L. multifora*). More species-level studies and studies on density compensation are needed to complement existing higher-level data. Nevertheless, the mismatches between *in situ* and laboratory data suggest that indirect ecological effects of OA may become important mechanisms of change, magnifying any direct physiological effects. They also show that our current understanding of which marine taxa will be adversely affected by OA is still far from comprehensive.

### (b) Loss of structural complexity and live coral as habitat

Our study documented reduced fine-scale structural complexity in coral reefs at high CO_2_, confirming the results of an earlier study [7]. The loss of habitat complexity was associated with severe losses in the densities, in particular, of some mobile groups (e.g. decapod crustaceans and crinoids), with their nocturnal behaviour suggesting high predation pressure by visually hunting fish. By contrast, sessile groups protected from predators by shells or toxins (bivalvia, ascidians), and mobile groups protected by shells (gastropods, hermit crabs), large sizes or toxins (some of the echinoderms, nudibranchs), were far less dependent on structural complexity. Our study also showed richest macroinvertebrate communities in quadrats occupied by living coral, suggesting that live corals represent essential habitat rather than competitors for space or food for many groups. The observed reduction in macroinvertebrates associated with the loss of structural complexity and live coral is of concern, because cover and structural complexity of coral reefs have been declining at an alarming rate around the world [16,42,43]. A substantial proportion of the decline is attributed to rising temperatures, OA, more frequent coral bleaching and intense storm events, and greater rainfall variability from increasing CO_2_ emissions [16,42,43].

**Figure 4.**
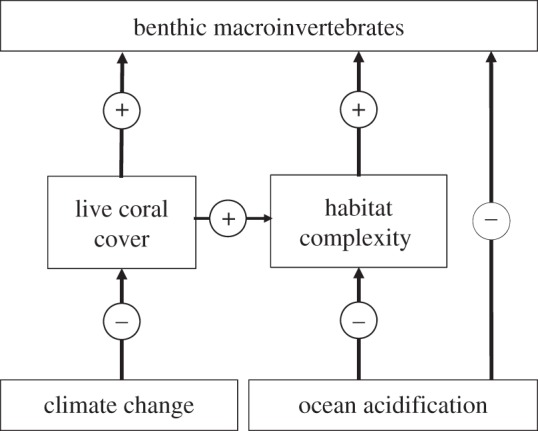
Conceptual diagram of the loss of coral cover owing to climate-change-related disturbances and the indirect and direct effects of OA on reef-associated macroinvertebrates.

In conclusion, our study shows that reef-inhabiting macroinvertebrates will be affected by several interrelated pathways in a high-CO_2_ world (figure 4). First, the loss of live coral cover owing to climate change and other anthropogenic disturbances [42,43] will cause severe losses not only to coral cover but also to reef-inhabiting macroinvertebrates. Second, as shown here, the loss of refugia and habitat is an important additional mechanism of how OA will affect macroinvertebrate communities. Third, as demonstrated in numerous experimental studies, OA will directly affect the physiology, calcification, reproduction, behaviour, neuronal functions and survival of many groups. As sensitivities to these disturbances vary between groups, we anticipate shifts in food webs, altered competitive advantages and functional replacements in reef communities. Thus, it is essential to combine empirical evidence from both controlled CO_2_ perturbation experiments and the field, to improve our understanding of the combined physiological and ecological mechanisms and thresholds. Despite incomplete knowledge, however, our data strongly suggest that unless urgent action is taken to prevent further substantial CO_2_ rises, the biodiversity of many benthic communities will continue to decline.

## Data accessibility

The data are available through the MEST Data Archive of the Australian Institute of Marine Science.
